# Eye Movement Desensitization (EMD) to reduce posttraumatic stress disorder-related stress reactivity in Indonesia PTSD patients: a study protocol for a randomized controlled trial

**DOI:** 10.1186/s13063-021-05100-3

**Published:** 2021-03-04

**Authors:** Eka Susanty, Marit Sijbrandij, Wilis Srisayekti, Anja C. Huizink

**Affiliations:** 1 Faculty of Psychology, Universitas Jenderal Achmad Yani, Cimahi, Indonesia; 2 Department of Clinical, Neuro and Developmental Psychology, Faculty Behaviour and Movement Sciences, Vrije Universiteit Amsterdam, Van der Boechorststraat 7, 1081 BT Amsterdam, The Netherlands; 3 Department of General and Experimental Psychology, Faculty of Psychology, Universitas Padjadjaran, Bandung, Indonesia

**Keywords:** Stress reactivity, Posttraumatic stress disorder, Eye Movement Desensitization and Reprocessing, HRV, PEP, Indonesia

## Abstract

**Background:**

Posttraumatic stress disorder (PTSD) may develop after exposure to a traumatic event. Eye Movement Desensitization and Reprocessing (EMDR) is an evidence-based psychological treatment for PTSD. It is yet unclear whether eye movements also reduce stress reactivity in PTSD patients. This study aims to test whether eye movements, as provided during Eye Movement Desensitization (EMD), are more effective in reducing stress reactivity in PTSD patients as compared to a retrieval-only control condition.

**Methods:**

The study includes participants who meet criteria of PTSD of the public psychological services in Jakarta and Bandung, Indonesia. One hundred and ten participants are randomly assigned to either an (1) Eye Movement Desensitization group (*n* = 55) or (2) retrieval-only control group (*n* = 55). Participants are assessed at baseline (T0), post-treatment (T1), 1 month (T2), and at 3 months follow-up (T3). Participants are exposed to a script-driven imagery procedure at T0 and T1. The primary outcome is heart rate variability (HRV) stress reactivity during script-driven imagery. Secondary outcomes include heart rate (HR), pre-ejection period (PEP), saliva cortisol levels, PTSD symptoms, neurocognitive functioning, symptoms of anxiety and depression, perceived stress level, and quality of life.

**Discussion:**

If the EMD intervention is effective in reducing stress reactivity outcomes, this would give us more insight into the underlying mechanisms of EMDR’s effectiveness in PTSD symptom reduction.

**Trial registration:**

ISRCTN registry ISRCTN55239132. Registered on 19 December 2017.

**Supplementary Information:**

The online version contains supplementary material available at 10.1186/s13063-021-05100-3.

## Administrative information

**Table:** 

**Title {1}**	Eye Movement Desensitization (EMD) to reduce posttraumatic stress disorder-related stress reactivity in Indonesia PTSD patients; a study protocol for a randomized controlled trial
**Trial registration {2a and 2b}.**	Item 2a: ISRCTN: 55239132, ISRCTN registry Item 2b: We added the table of WHO trial registration data set as an additional file
**Protocol version {3}**	19 December 2017 – 7 July 2018: Original version 7 July 2018 – 31 March 2019: Amendment 01. Primary reason for amendment Change in section 11a, regarding step of EMDR intervention. We excluded installation phase of EMDR procedure. Amendment 02. Primary reason for amendment. Change in section 1. We also changed the title of trial as consequence deleting installation phase. We used EMD term for the name of intervention. 1 April 2019 – now: the last version
**Funding {4}**	Indonesian Endowment Fund for Education (LPDP) Ministry of Research Technology and Higher Education cooperate with Ministry of Finance, Republic of Indonesia through Beasiswa Unggulan Dosen Indonesia (BUDI LN) has provided for funding for the research. The following website address: https://www.lpdp.kemenkeu.go.id/
**Author details {5a}**	**Author details** ^1^Faculty of Psychology, Universitas Jenderal Achmad Yani, Cimahi, Indonesia, ^2^Clinical, Neuro and Developmental Psychology, Faculty of Behavioral and Movement Science, Vrije Universiteit Amsterdam, Netherland, ^3^ Department of General and Experimental Psychology, Faculty of Psychology, Universitas Padjadjaran, Bandung, Indonesia. **Authors’ Contributions** ES, MS, WS and AH, work on the original idea of this study and developed design. MS gave detail advice for each of subtitle of protocol specify on the EMD protocol in this study. AH gave advice for each of subtitle of protocol specify on physiological measurement. WS gave input on methodology, ethical issue, and specify term of protocol. All authors contributed to this trial protocol paper and approved of the final version of the manuscript
**Name and contact information for the trial sponsor {5b}**	sponsor-investigator, Eka Susanty, e.s.susanty@vu.nl, Departement of Clinical, Neuro and Developmental Psychology, Faculty of Behavioral and Movement Science, Vrije Universiteit Amsterdam, Van der Boechorststraat 7, 1081 BT Amsterdam, The Netherlands
**Role of sponsor {5c}**	This funding source had no role in the design of this study and will not have any role during its execution, analyses, interpretation of the data, or decision to submit results

## Background

In Indonesia, people are at high risk for trauma exposure, including natural disasters, violent crime, sexual harassment, or experiencing a severe accident, which may lead to posttraumatic stress disorder [1, 2]. Therefore, it can be expected that a high prevalence of PTSD may occur in Indonesia. Research on PTSD in Indonesia is limited. According to existing studies, the prevalence rate of PTSD was 47% among adolescents, 6 months following an earthquake in Aceh [3]. Another study by Down et al. found that overall PTSD rate after major natural disasters in Sumatera and Java was 20.9% [4].

Several psychological interventions are applied to deal with PTSD symptoms. The strongest empirical evidence exists for trauma-focused Cognitive Behavioral Therapy (TF-CBT) and Eye Movement Desensitization and Reprocessing (EMDR) [5, 6]. In this study, we focus on EMDR, more specifically, on the first part of this treatment: Eye Movement Desensitization (EMD). Some preliminary evidence exists for the effect of EMDR on stress measures [7, 8]. It is of interest to study how EMD can get under the skin of patients and alter the level of stress reactivity in PTSD patients.

The effect of eye movements in EMDR therapy process has been explained through the working memory theory, which states that both recalling an emotional memory and making eye movements simultaneously, requires working memory capacity. Performing this dual-task requires working memory capacity and renders the memory less vivid and less emotional [9]. Support for this theory has been found in several experimental studies in healthy samples that reported lower vividness and emotionality of negative autobiographic memories after recall with eye movements (e.g., [10, 11]). Although these studies have shown that EMs significantly reduce memory vividness and emotionality of aversive memories, some limitations remain. The majority of these studies were done in non-clinical healthy student samples. Therefore, the results are difficult to translate to clinical PTSD populations (e.g., [12, 13]).

In addition, only a few studies examined whether EMDR improves stress reactivity related to traumatic memory retrieval as part of the arousal and activity cluster of symptoms in PTSD [14]. Stress reactivity is defined as the reactivity of the autonomic nervous system, which is represented by decreased heart rate variability (HRV) and decreased pre-ejection period (PEP), and increased heart rate (HR) in response to a trauma-related stressor [15]. It has been suggested that EMs in EMDR are beneficial and have a distinct effect on psychophysiological responding [16]. The orienting response theory of EMDR states that EMs, or other dual-attention stimuli, elicit an orienting response with associated physiological de-arousal that enhances trauma material processing [17]. The previous studies in non-clinical and clinical participants have found that EMDR led to significant reductions in heart rate and skin conductance responses to trauma recall compared to a relaxed state [16, 18].

Therefore, it is important to examine whether EMD is associated with reduced stress reactivity among clinical PTSD patients. Psychophysiological stress mechanisms that may play a role in PTSD treatment effects include reduced physiological stress reactivity, assessed with heart rate (HR) and heart rate variability (HRV) responses, and neuroendocrine stress response, as indexed by cortisol levels in saliva [19, 20]. HRV represents the change in the time interval between successive heartbeats. HRV provides an index of the parasympathetic nervous system and has been associated with emotional self-regulation [21].

Besides activating autonomic functioning, stress also activates the hypothalamic-pituitary adrenal (HPA) axis. Activation of the HPA axis is indexed by heightened cortisol levels. High levels of saliva cortisol have been associated with general life stress and emotional regulation that influences cognitive function [22, 23].

A previous study has found a significant relationship between PTSD symptoms and neurocognitive deficits, suggesting that PTSD symptoms may also interfere with executive function and may cause impairments in cognitive flexibility [24]. Another study showed that improved neurocognitive functioning might be a potential mechanism for long-term maintenance of treatment gains following CBT for PTSD [25]. This study will focus on specific elements of neurocognitive functioning, including memory, attention, and information processing, and examine whether these indices improve after EMD.

If we find evidence for reduced stress reactivity and improved neurocognitive functioning after EMD, it can help in understanding part of the underlying mechanisms through which EMD exerts its effects on PTSD symptom reduction. To examine this, we will compare EMD with a control condition, consisting of the same procedure as EMD, but without the EM. Furthermore, we will investigate whether PTSD symptom reduction is related to reduced stress reactivity and neurocognitive functioning. We hypothesize that participants with PTSD receiving the EMD condition will show larger stress reactivity reductions in response to trauma scripts and greater improvements in neurocognitive functioning than the retrieval-only control group.

## Objective

This study aims to test whether particularly Eye Movement Desensitization (EMD), the first part of EMDR treatment, is more effective in reducing stress reactivity in PTSD patients, as compared to a retrieval-only control condition. The current study also investigates whether EMD is related to improved neurocognitive function, including memory, attention, and information processing.

## Methods

### Design

**Fig. 1:**
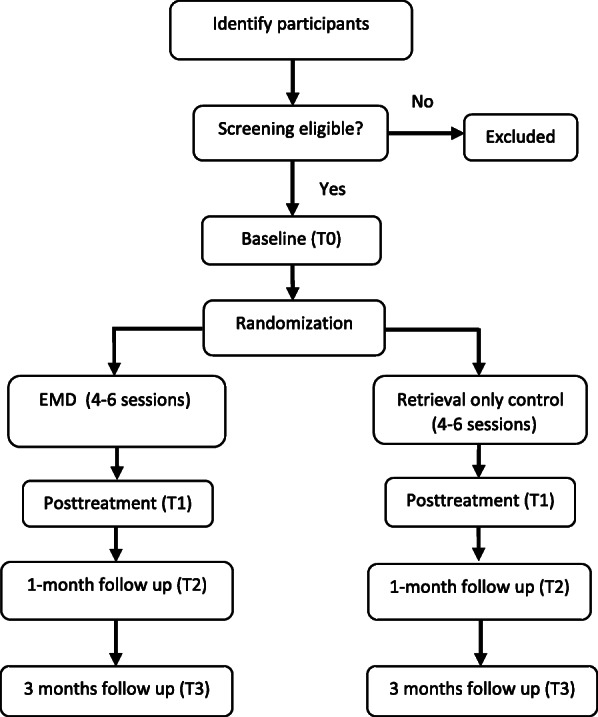
Flow chart of the trial protocol

Figure 1 shows the randomized clinical trial (RCT) design of this study (see Fig. 1, flow chart of the trial protocol). Assessments during this study will be conducted during baseline (T0), 1-week post-treatment (T1), 1-month follow-up (T2), and 3 months after treatment (T3). The study has been approved by the research Ethics Committee Universitas Padjadjaran Bandung on 2 July 2018. Document number: 3 35/UN6.KEP/EC/2018.

### Inclusion and exclusion criteria

Participants will be included when they meet the following criteria: (1) Diagnostic and Statistical Manual of Mental Disorders, fifth edition (DSM-5) diagnosis of PTSD as diagnosed with the Structured Clinical Interview for DSM-5 disorders (SCID-5) and (2) age of 18 years or older. Participants will be excluded if they meet any of the following criteria (as indicated by SCID-5): (1) current or previous psychotic disorder, (2) current substance use disorder, (3) acute suicidality, and (4) current organic disorder, i.e., epileptic and brain damage.

### Recruitment

Recruitment of participants will take place from 21 April 2019 to 31 December 2020. Participants (*N* = 92) will be recruited at the public psychological services of Jakarta, Bandung, and Cimahi. These public centers are (1) the “Pulih” clinic in Jakarta, (2) the “psychology service Unisba” in Bandung, and (3) the “crisis center Unjani” in Cimahi. Recruitment will be performed by public announcements through brochures, social media (Facebook, a WhatsApp group, and Instagram), and on the website of the Unjani and “Pulih” clinics. Potential participants who are interested in the project can contact the research team by phone. Then, the research team will perform a first eligibility check by phone.”

### Procedure

#### Screening

The study is open for both clients who are already a patient in one of the participating centers and new patients. Both new, chronic, and recurrent PTSD patients are included, and for all participants, the inclusion criterion is a current DSM-5 diagnosis of PTSD. The candidate participant will be invited to the clinic and asked for oral and written informed consent. Next, the participant will be administered the SCID-5 by a trained assessor. The following self-report questionnaires will also be administrated: sociodemographic questions (i.e. gender, age, ethnic), medical history (i.e. medical intervention, kind of drug consumed), and the PCL-5 (PTSD Checklist for DSM-5) [26]. If a candidate meets the inclusion criteria and none of the exclusion criteria, the participant will be invited to the next step of the baseline assessment after a break of 15 min.

A research assistant will provide information about the purpose of the study, including the background rationale of the study, risks and safety, benefits, and their right to withdraw from the study at any time without consequences. The participants will be asked oral and written informed consent for the RCT before entering the study.

The research assistant will continue the baseline assessment by administering the Hopkins Symptoms Checklist-25 (HSCL-25) [27], the Perceived Stress Scale (PSS [28];), and the World Health Organization Quality of Life (WHOQOL-BREF [29];). Neurocognitive tests administered at baseline include the Trail Making Test (TMT) [30], California Learning Verbal Test (CLVT [31];), and Subtest Digit Span of the Wechsler Adult Intelligence Scale fourth edition (WAIS-IV) [32]. Finally, participants will be instructed on how to collect saliva for cortisol analyses.

The second part of the baseline assessment includes the physiological measurements, using the VU University Ambulatory Monitoring System (VU-AMS) device when the participant listens to the traumatic script. The physiological measurements are performed 1 week after the T0, directly before the first treatment session. The reason is that the traumatic script needed for the physiological measurements will be prepared based on the information gathered at the T0. The recording procedure of VU-AMS is explained in the stress measurement section.

#### Randomization

After all baseline assessments have been completed, randomization will be conducted. We use the randomization tool that is included in the Castor data management software (www.castoredc.com). Participants will be allocated in a 1:1 using block randomization into one of two conditions: (1) EMD and (2) retrieval-only control which consists of the EMD protocol without the EMs. Block sizes of 4, 6, and 6 will be selected randomly within the randomization process. The time span between T0 and the first intervention session will be approximately 1 week.

At post-treatment after the final session, the PCL-5, HSCL-25, PSS, WHOQOL-BREF, TMT, CVLT, Digit Span, HRV/HR, PEP, and saliva collection for cortisol will re-administered. Then, 1 month (T2) and 3 months (T3) after the final session, the PCL-5, HSCL-25, PSS, WHOQOL-BREF, TMT, CVLT, and digit span WAIS-IV subtest will re-administered. The follow-up assessment will be performed in two steps. First, participants will be contacted personally and motivated to complete the follow-up assessment. In case participants refuse, they will be offered an assessment of primary outcome only. If participants still refuse, they will be asked to provide reasons for their refusal, which will be documented.

#### Blinding

The study is single blind. Participants will be informed about the condition they are assigned to. The research coordinator and therapist will not be blinded to treatment allocation. The assessors are blinded, meaning that an assessor is not aware of the condition to which the participant is assigned. The statistical analysis will be performed by statistician who is blinded to treatment allocation. The statistician is a doctoral researcher who is an expert in data analysis using R statistics software.

#### Stress measurement

Physiological responses to scripts (see script-driven imagery [33];) are acquired by using the VU-AMS device, which records the electrocardiogram (ECG) and the impedance cardiogram (ICG) continuously through seven disposable electrodes. The reliability and validity of the VU-AMS device have been reported as adequate [34]. Stress reactivity will be measured through HR/HRV and PEP variables. HRV and pre-ejection period show variability and are indicators of cardiac autonomic nervous system activity (sympathetic drive by pre-ejection period, and parasympathetic drive by HRV) [35].

**Fig. 2:**
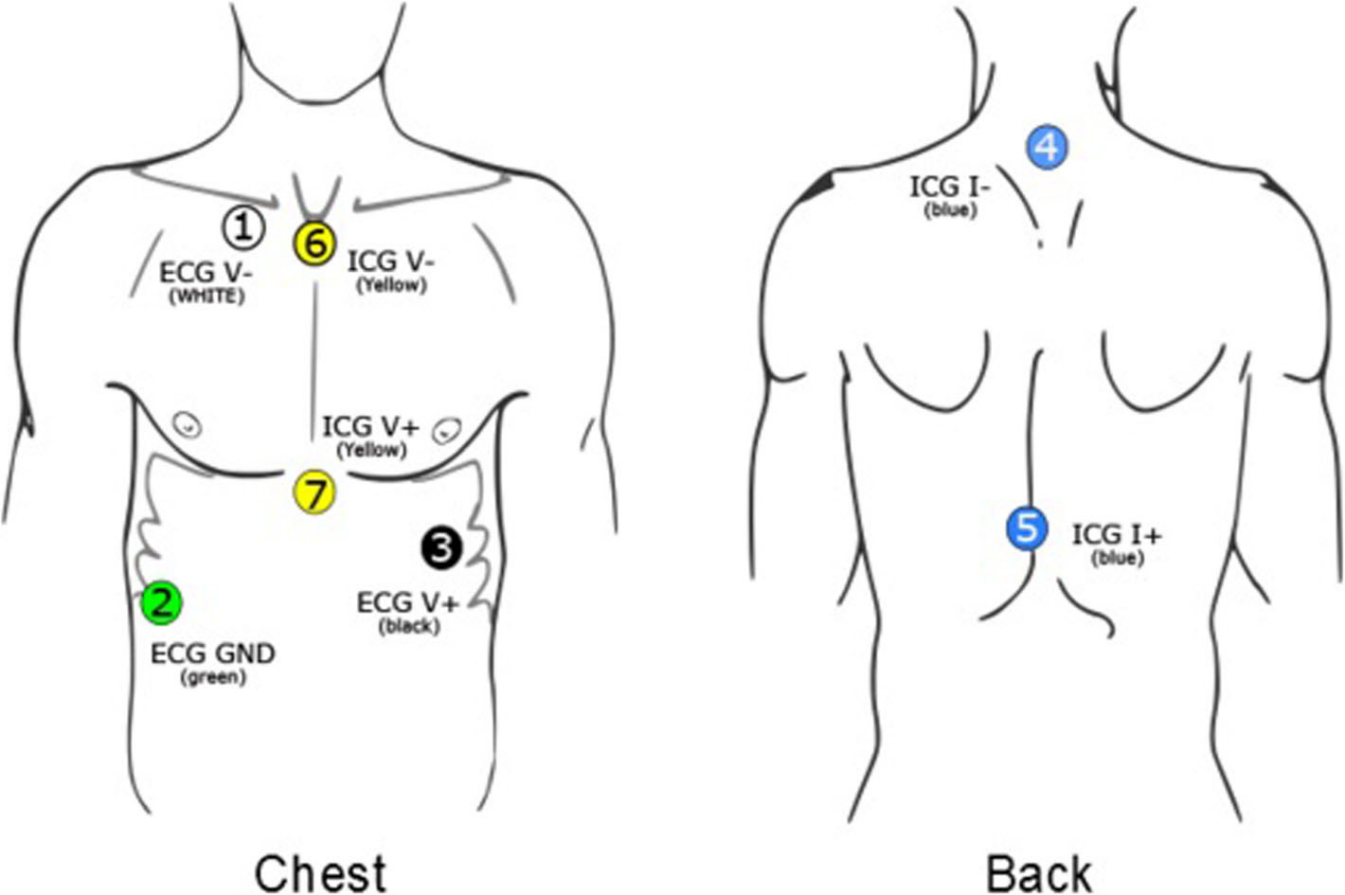
Lead electrodes placement

The recording process will be held in a private area or any location that is relatively quiet and free of noise. The participants will be seated in a comfortable chair, and their feet should be able to reach the floor. The participants should not do the following activities at least 1 h prior to the recording: heavy aerobics, consumption coffee, tea, or other caffeine. No cigarettes should be smoked from 30 min before the recording. Participants are asked to wait at least 1½ h after a heavy meal to take the measurement. Current medications are recorded. Research staff will explain to participants that the goal of this session is to gather information about stress responses to trauma scripts. They also will be informed that excessive movement may result in artifacts and therefore, they are asked sit quietly without talking, falling asleep, crossing legs, or making unnecessary movements. It is preferable that participants keep their eyes open. The test leader will place 7 electrodes on the surface of the participant’s skin. After attaching the electrodes, the test leader checks the signal on the monitor screen. If the signal looks stable, then the recording process is ready to begin (see Fig. 2, lead electrodes placement).

#### Script-driven imagery

Stress responses to neutral and traumatic stimuli will be measured using the script-driven imagery procedure [33]. For the current study, the researcher prepared “visiting the national library” as a neutral script. Personalized traumatic scripts will be made based on of participants’ trauma narratives. The participants are first asked to describe their traumatic event in detail, which the interviewer will write down in about 450–500 words. The story includes a mental image of the traumatic experience that produces distress. This traumatic script will be read and audio-recorded by an independent female person. The recordings of the neutral and trauma scripts will be played during physiological stress assessment using the VU-AMS device [36].

#### Physiological recording using VU AMS

**Fig. 3:**
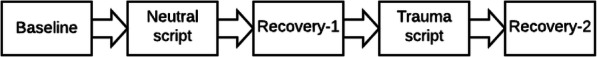
Timeline of VU-AMS recording

The total physiological stress measurement procedure will include five stages, during which stress indices, including HRV/HR and PEP, will be assessed using the VU-AMS device. The five stages are as follows: baseline, neutral script, recovery-1, traumatic script, and recovery-2. The duration of each stage is 5 min (see Fig. 3, timeline of VU-AMS recording). At the baseline, the participant is seated in a relaxed manner without doing any activities. Similarly, at recovery-1 and recovery-2, the participant can relax, and will be allowed to read provided magazines. At the neutral script stage, the participant will be asked to listen carefully to the script “visiting the national library” for 3 min. This stage lasts for 5 min and will continue to recovery-1 stage lasts for 5 min. In the following stage, participants will be asked to listen carefully to the trauma script and then to re-experience the emotions or feelings associated with trauma events for 3 min. This stage lasts for 5 min. Finally, the last stage of recording is recovery-2 period of 5 min. This process will be conducted directly before (session 1) and after treatment (session 6/last intervention).

#### Cortisol assays protocol

Cortisol levels will be determined in saliva using salivette sampling devices by the competitive immunoenzymatic method [37]. This method is used for quantitative determination of cortisol concentration in saliva. The area under the curve concerning for to the ground (AUCg) will be determined, as well as the cortisol awakening response (CAR) [38]. Participants are instructed to collect the first sample immediately after waking up or at 7 am, at 30-min post-waking, at 12.00 pm, and at 8.00 pm. Samples are stored in participants’ freezers until research staff collects them to stores them in a freezer at − 20 °C until taken to the laboratory for assay (maximum storage 6 months).

### Outcome measures

#### Primary outcome

The primary outcome of this study is the level of the HRV in response to the trauma script. HRV is an indicator of the activity of the parasympathetic nervous system, which is active in resting conditions. HRV represents the change in the time interval between successive heartbeats [21]. The time between beats is measured in milliseconds (ms) and is called an R-R interval or inter-beat interval (IBI). Time domain analyses measures the changes in heart rate over time or the intervals between successive normal cardiac cycles. We will use root mean square of the successive differences (RMSSD) as the outcome metric for the primary outcome. RMSSD is one of a few time-domain tools used to assess HRV, the successive differences being neighboring RR intervals [39]. Kim and Woo found that normal range of RMSSD in population was 29.7 ± 18.1 ms [40]. In this study, we expect that there is a difference in the change of HRV (Δ HRV) between the experimental and the control group (Δ HRV in the experimental group is smaller than Δ HRV in the control group). HRV will be measured at time point T0 (baseline) and T1 (post-treatment) for all participants.

### Secondary outcomes

#### Heart rate (HR)

Heart rate, a general index of arousal due to for examples stressful conditions, is influenced by both the parasympathetic and the sympathetic nervous system. Heart rate is usually measured in beats per minute (bpm), using the R-peak of the QRScomplex as a marker. The time between two R-peaks is measured over a given period of time, resulting in a RR-interval (or NN-interval, referring to normal beats). We will compute the level of HR in response to the trauma script.

#### Pre-ejection period (PEP)

The pre-ejection period (PEP) is defined as the time interval from the beginning of electrical stimulation of the ventricles to the opening of the aortic valve. PEP is an indicator of the sympathetic nervous system activity, which is high under stressful conditions [35]. We will compute the level of PEP in response to the trauma script.

#### Other measures

The SCID-5 is a semi-structured interview for DSM-5 Axis I disorders [41]. In this study, we administer three modules during screening: the Trauma and Stressor-Related Disorder to diagnose PTSD, Psychotic and Associated Symptoms, and Substance Use Disorders Modules, using the Bahasa Indonesian version of the SCID-5 [42].

Details of assessment instruments and time points are found in the Standard Protocol Items: Recommendations for International Trial (SPIRITS) Checklist (see Table 1). Other symptoms (PCL-5, HSCL-25, PSS) and quality of life (WHOQOL-BREF) will be measured at every time point at T0 (baseline), T1 (post-treatment), T2 (1 month follow-up), and T3 (3 months follow-up). We also administer the PCL-5, HSCL-25, PSS, and WHOQOL-BREF after a session of treatment.

**Table 1:**
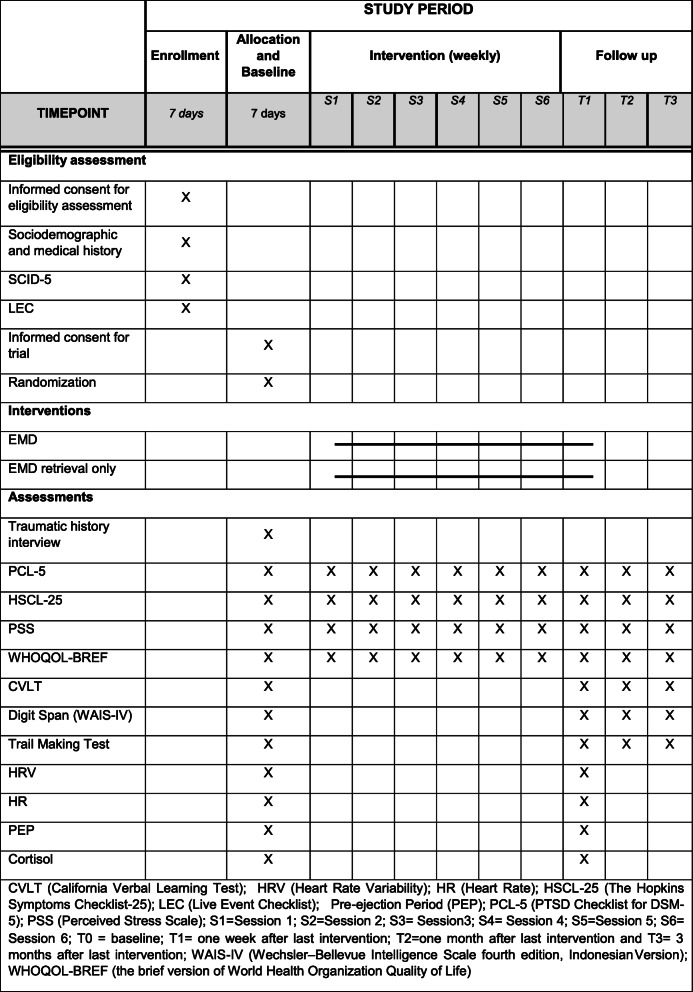
The schedule of enrollment, interventions, and assessments

The PCL-5 is a self-report 20-items questionnaire to assess the 20 DSM-5 symptoms of PTSD on a scale from 0 to 4 (“not at all (0)” to “extremely (4)”). Items are summed to provide a total severity score and total score range between 0 and 80. PCL-5 scores exhibited strong internal consistency (*α* = 0.94), test-retest reliability (*r* = 0.82), and convergent (rs = 0.74 to 0.85) and discriminant (rs = 0.31 to 0.60) validity in a study in trauma-exposed college students [43]. We use the Indonesian version of the PCL based on the DSM-5 [44].

The Live Events Checklist (LEC) will be used to assess which traumatic event(s) participants have experienced. The LEC has strong convergence with measure of psychopathology that are known to be associated with trauma exposure [45]. The LEC assesses exposure to 16 events known to potentially result in PTSD or distress and includes one additional item assessing any other extraordinarily stressful event. The participants rate their experience of the events on a 5 point nominal scale (1 = happened to me, 2 = witnessed it, 3 = learned about it, 4 = not sure, and 5 = does not apply), and they may endorse multiple levels of exposure to the same trauma type. The LEC does not yield a total score or composite score.

The Hopkins Symptoms Checklist-25 (HSCL-25) is used to measure anxiety and depression symptoms. The HSCL-25 consists of two parts: anxiety symptoms (10 items) and depression (15 items). Symptoms are scored on a five-point scale varying from “not at all (0)” to “extremely (4),” and total scores range between 0 to 40 for anxiety and 0 to 60 for depression, with higher scores indicating more symptoms. The HSCL-25 has been translated and culturally adapted for use in Indonesia [46]. The HSCL-25 is reliable and valid across a variety of cultural groups, including Indonesia [47].

The Perceived Stress Scale (PSS) is the most widely used instrument for measuring perceived feelings and thought of stress during the last month [28]. The PSS is one of the most widely used and established validity and reliability [48]. The PSS consists of 10 items on a scale from 0 to 4 (0 = never, 1 = almost never, 2 = sometimes, 3 = fairly often, 4 = very often). The PSS has been translated into Indonesia language. The reliability measure for PSS-10 using the Cronbach alpha was 0.84 [49].

Quality of life will be assessed using the World Health Organization Quality of Life (WHOQOL-BREF) [29]. The instrument consists of four domain scores and two other items measuring the overall perception of quality of life and general health during the past 4 weeks. The domains are grouped into physical health (7 items), psychological (7 items), psychological health (6 items), social relationships (3 items), and environment (8 items). Domain scores are scaled in a positive direction (1 = not at all, 2 = not much, 3 = moderately, 4 = a great deal, 5 = completely). The scores are transformed into a linear scale between 0 and 100, with lower scores indicating lower levels of quality of life. The WHOQOL-BREF has showed adequate psychometric properties across many contexts and across many health conditions in many countries [50, 51]. We will use Indonesian version of the WHOQOL-BREF [50].

#### Neurocognitive functioning

The neurocognitive instruments will be administered at baseline (T0), post-treatment (T1), one month (T2), and 3 months follow-up (T3). Wechsler Adult Intelligence Scale-Fourth Edition (WAIS-IV) has been validated and is commonly used embedded performance validity tests (PVTs) [52]. The WAIS-IV-ID (Indonesian version) has been adapted and validated by Suwartono et al (2014) [53, 54]. To assess attention and working memory, we administer the Digit Span subtest, a 3-part test involving digit span forward, backward, and sequencing [55]. Digit span forward is a test involving, attention, encoding, and auditory processing. Digit span backward will measure working memory, transformation of information, mental manipulation, and visuospatial imaging. Digit span sequencing was designed to measure working memory and mental manipulation of information.

The California Verbal Learning Test (CVLT) is a five-trial learning task, followed by an interference list and short delay free recall which measures encoding, short-term retrieval, and recognition of verbal information [56]. Participants are asked to recall a number of words in A list (immediate free recall), words in B list (immediate free recall), and last words in A list (short delay free recall) [57]. The CVLT second edition was translated and culturally adapted to Indonesian Multiple Sclerosis patients [58]. Estiasari et al. reported CVLT is valid and reliable and can be widely used to assess cognitive function in Indonesian participants [58].

The Trail Making Test (TMT) measures information processing speed, shift of attention, planning, and cognitive flexibility [59]. The TMT comprises of a part A and part B. In part A, the participant uses a pencil to connect a series of 25 encircled numbers in numerical order. In part B, the participant connects 25 encircled numbers and letters in numerical and letters. The administration time will generally range from 5 to 10 min [30]. The study results suggest that TMT A and B demonstrated adequate validity and reliability for assessing executive function and visuospatial processing speed among older adults [60, 61].

### Intervention

#### EMD treatment protocol

The procedures of EMD intervention will be carried out in line with the standard EMDR protocol [62]. Since we aimed to evaluate the effects of eye movement during retrieval of a traumatic memory, we decided to omit the installation phase from the original EMDR procedure in both study groups. It has been suggested that the installation phase may be counter-effective, since performing eye movements when retrieving a positive cognition or image (as done in the installation phase) may render that positive image less vivid and positive [9]. EMD will be given during 4 to a maximum of 6 sessions, and each session lasts 45–60 min. The therapist will provide at least 4 sessions, even if their Subjective Units Distress (SUDs) scores decrease to 0 or 1 in less than 4 sessions and stop when SUDs = 0 or 1 for all target memories. If the participant does not reach 0 or 1 in 6 sessions, the therapy will be ended nevertheless, to minimize heterogeneity. In addition, reductions in distress may also take place after the therapy has ended. SUDs measure the level of distress before and after target memory processing, where 0 is no disturbance or neutral and 10 is the highest disturbance.

EMD consists of the following steps: *(1) Client History* and *treatment planning*: obtaining information regarding the clients’ clinical condition, including intrusive emotions and physical sensations. *(2) Preparation*: building a therapeutic bond with the client, the explanation of EMDR process and its effects. *(3) Assessment*: identification of the target visual image of the traumatic memory and associated negative emotions. The participant describes the intensity of the negative emotions on a 0–10 SUDs scale). *(4) Desensitization*: clients will be asked to focus on target traumatic events, while focusing their eyes on the therapist’s finger that moves from left to right an back in the participant’s visual field. The therapist will conduct EM for 24 cycles several times. This phase will end if SUD scores reach 0 or 1. Next, participants will scan their body until any tension disappears. *(5) Closure*: the session is closed, and the stabilization techniques and relaxation exercises are reviewed. Sessions 2–4 will start with a reevaluation of the patient’s progress and SUD scores of target events to guide the choice of continuing with the target traumatic event or choosing a new event.

The EMD treatments will be performed by experienced psychotherapists with at least 1 year of experience in treating PTSD patients. Eight therapists are recruited through colleagues from the Clinical Psychologist Association (IPK). An accredited EMDR supervisor will supervise therapists weekly.

#### Retrieval-only condition (control)

Control participants will receive the same treatment as the EMD group participants, except that during phase *(4) Desensitization*, no eye movements will be performed during retrieval of the trauma memory.

#### Data collection, quality control, and confidentiality

All data will be pseudo-anonymized. A coding list with participant numbers and names, and the informed consent forms, will be stored in a separate locked place at the research coordinator’s office. Data are stored in the Castor Data Management program.

Drop out and premature termination from study or treatment at any point after randomization will be recorded. The participants can choose to withdraw from the trial intervention, withdraw from follow-up, withdraw from both conditions, and ask that previously collected data not be used. A participant may be withdrawn from intervention if he/she feels does not fit into the given intervention technique (e.g., the participant feels nauseous doing eye movements task when recalling the memory). Participants who withdraw from the intervention as part of the trial will proceed to the follow-up assessment unless they withdraw consent for this. For any participant reluctant to complete the full outcome assessment at follow-up, we will attempt to obtain the PCL-5, PSS, HSCL-25, WHOQOL-BREF, CVLT, Digit Span (WAIS-IV), and TMT. As much information as possible will be collected from protocol non-adherents including reasons for non-adherence. All randomized participants will be included in the intention to treat analysis.

#### Protection and assessment of safety

The study has been approved locally by the Health Research Ethics Committee of Medical Faculty of Padjadjaran University (KEPK-FK Unpad) on 26 June 2018. Adverse events (AEs) will be reported to KEPK-FK Unpad by the principal investigator.

#### Sample size

We expect that HRV will decrease in both groups at T1 compared with T0, with a smaller decrease in the EMD group, resulting in greater HRV at T1 in the EMD group compared with the EMD retrieval-only control group. No studies have been carried out yet comparing the effect of EMD to retrieval only on HRV in PTSD patients to inform power calculations. However, based on the theoretical assumptions and studies with other related outcome measures [63, 64], we expect to detect a difference between the EMD condition and retrieval only on HRV of a medium effect size (Cohen’s *d* = 0.4). Power calculations were conducted using G*power software and were based on based on a general linear model (GLM). Linear mixed-effect model can be conceptualized as GLM (repeated measure ANOVA) as a balanced design within independent sampling units, and an unstructured covariance model is assumed [65]. We transformed for Cohen’s *d* = 0.4 into *f* = 0.2. For a 2 between (treatment EMD conditions) X 2 within (baseline, post-treatment) repeated measure ANOVA (*α* = 0.05, power = 0.9, correlation between measures = 0.4, and medium effect-size *f* = 0.2) [7, 66]. A power calculation suggested that a total sample size of *N* = 82 will be required, which is 41 per condition. All power computations were conducted in the G*Power software. Considering drop out of participants, we will recruit an additional 25% extra participants, yielding a total number of 55 participants per condition, thus 110 participants in total.

#### Statistical analysis

Baseline sociodemographic and outcomes of interest are first compared across treatment conditions. Data will be described by reporting results from general descriptive analysis. The categorical variables will be summarized as numbers and percentages and continuous variables as means and standard deviations, minimum, and maximum.

Following this, intention-to-treat analysis will be performed only for the primary outcome, and then performing multiple imputation with a sensitivity analysis using both observed and imputed data. *P* values of < 0.05 will be considered significant.

Statistical analysis will be conducted using R Statistics software. The stress reactivity outcome variables were measured only at T0 and T1. HRV is a primary outcome that is a continuous dependent variable. We will analyze our data using repeated measures analysis of variance (ANOVA) to examine the interaction between time (pre-post intervention), within-subject factor, and the between-group factor treatment group. This analysis allows us to compare the two groups over time to examine whether participants in the EMD condition benefit more in terms of stress reduction, when compared to the retrieval-only condition, from baseline to post-treatment.

We will analyze the secondary outcomes (self-report and neurocognitive measures) using linear mixed models, with a random effect for participants. Data from time points T0, T1, T2, and T3 will be included in the repeated measures models. In all analyses, a treatment × time interaction term will represent the effect of EMD and EMD retrieval-only interventions of the outcome variables over time. Trauma type, gender, and age will be included as covariates in all models, both for the primary as well as the secondary outcome(s).

## Discussion

RCTs have proven that PTSD treatment using EMDR is effective in reducing PTSD symptoms [5, 6]. This study will compare eye movements to retrieval only to understand the role of EMs within EMD with regard to its effect on stress reactivity and neurocognitive functioning in response to trauma scripts in Indonesian patients diagnosed with PTSD. If the EMD intervention is proven effective in terms of stress reactivity reduction, improved neurocognitive functioning and the reduction of PTSD symptoms shortly after the intervention up to 3 months follow-up, it would give us more insight into the underlying mechanisms of EMD as intervention. This would provide empirical evidence that the additional effect of EMs will extend to stress reactivity measures and may further support the contribution of eye movements and similar dual stimulation tasks to the effectiveness of EMDR treatment.

## Trial status

Protocol (ISRCTN55239132) was registered on 19 December 2017 and recruitment started on 21 April 2019. Recruitment will continue until 31 December 2020.

## Supplementary Information

**Additional file 1.**

**Additional file 2.**

**Additional file 3.**

**Additional file 4.**

**Additional file 5.**

**Additional file 6.**

**Additional file 7.**

**Additional file 8.**

## Data Availability

The datasets used and/or analyzed during the current study are available from the corresponding author on reasonable request.

## References

[1] Meutia I, Sofyan H, Schouler-ocak M (2018). Exposure to traumatic events and PTSD in a postconflict and disaster-prone area. J Loss Trauma.

[2] Sugiyanto G (2017). The cost of traffic accident and equivalent (case study in Indonesia). ARPN J Eng Appl Sci.

[3] Marthoenis M, Nirwana A, Fathiariani. Prevalence and determinants of posttraumatic stress in adolescents following an earthquake. Indian J Psychiatry 2019;61:526–528. 10.4103/psychiatry.IndianJPsychiatry_35_19 PMC6767826 31579181

[4] Downs LL, Rahmadian AA, Noviawati E, Vakil G, Hendriani S, Masril M (2017). A DSM comparative study of PTSD incidence in Indonesia. Adv Soc Sci Res J.

[5] Bisson JI, Ehlers A, Matthews R, Pilling S, Richards D, Turner S (2007). Psychological treatments for chronic post-traumatic stress disorder. Br J Psychiatry.

[6] Seidler GH, Wagner FE. Comparing the efficacy of EMDR and trauma-focused cognitive-behavioral therapy in the treatment of PTSD: a meta-analytic study. Psychol Med. 2006;36(11):1515–22. 10.1017/S0033291706007963 16740177

[7] Sack M, Hofmann A, Wizelman L, Lempa W (2008). Psychophysiological changes during EMDR and treatment outcome. J EMDR Pract Res..

[8] Sack M, Lempa W, Lamprecht F (2007). Assessment of psychophysiological stress reactions during a traumatic reminder in patients treated with EMDR. J EMDR Pract Res.

[9] van den Hout MA, Engelhard IM. How does EMDR work?. Journal of Experimental Psychopathology. 2012;3(5):724–38. 10.5127/jep.028212.

[10] van den Hout M, Muris P, Salemink E, Kindt M. Autobiographical memories become less vivid and emotional after eye movements. Br J Clin Psychol. 2001;40(2):121–30. 10.1348/014466501163571 11446234

[11] Leer A, Engelhard IM, van den Hout MA (2014). How eye movements in EMDR work: changes in memory vividness and emotionality. J Behav Ther Exp Psychiatry.

[12] van Schie K, van Veen SC, Engelhard IM, Klugkist I, van den Hout MA. Blurring emotional memories using eye movements: individual differences and speed of eye movements. Eur J Psychotraumatol. 2016;7(1):29476. 10.3402/ejpt.v7.29476 PMC4933794 27387843

[13] van den Hout M, Bartelski N, Engelhard IM (2013). On EMDR: Eye movements during retrieval reduce subjective vividness and objective memory accessibility during future recall. Cogn Emot.

[14] Lee CW, Cuijpers P (2013). A meta-analysis of the contribution of eye movements in processing emotional memories. J Behav Ther Exp Psychiatry.

[15] Gurel NZ, Carek AM, Inan OT, Levantsevych O, Abdelhadi N, Hammadah M (2019). Comparison of autonomic stress reactivity in young healthy versus aging subjects with heart disease. PLoS One.

[16] Schubert SJ, Lee CW, Drummond PD (2011). The efficacy and psychophysiological correlates of dual-attention tasks in eye movement desensitization and reprocessing (EMDR). J Anxiety Disord.

[17] Söndergaard HP, Elofsson U (2008). Psychophysiological studies of EMDR. J EMDR Pract Res.

[18] Schubert SJ, Lee CW, Drummond PD (2016). Eye movements matter, but why? Psychophysiological correlates of EMDR therapy to treat trauma in Timor-Leste. J EMDR Pract Res..

[19] Pacella ML, Feeny N, Zoellner L, Delahanty DL (2015). The impact of PTSD treatment on the cortisol awakening response. Depress Anxiety.

[20] Elsesser K, Sartory G, Tackenberg A (2004). Attention, heart rate, and startle response during exposure to trauma-relevant pictures: a comparison of recent trauma victims and patients with posttraumatic stress disorder. J Abnorm Psychol.

[21] Laborde S, Mosley E, Thayer JF. Heart rate variability and cardiac vagal tone in psychophysiological research–recommendations for experiment planning, data analysis, and data reporting. Front Psychol. 2017;8:213. 10.3389/fpsyg.2017.00213 PMC5316555 28265249

[22] Het S, Schoofs D, Rohleder N, Wolf OT (2012). Stress-induced cortisol level elevations are associated with reduced negative affect after stress: indications for a mood-buffering cortisol effect. Psychosom Med.

[23] Sroykham W, Wongsawat Y (2019). Effects of brain activity, morning salivary cortisol, and emotion regulation on cognitive impairment in elderly people. Medicine (Baltimore).

[24] Mozzambani ACF, Fuso SF, Malta SM, Ribeiro RL, Pupo MC, Flaks MK (2017). Long-term follow-up of attentional and executive functions of PTSD patients. Psychol Neurosci.

[25] Scher CD, Suvak MK, Resick PA. Trauma cognitions are related to symptoms up to 10 years after cognitive behavioral treatment for posttraumatic stress disorder. Psychol Trauma. 2017;9(6):750. 10.1037/tra0000258 PMC5550371 28182457

[26] Bovin MJ, Marx BP, Weathers FW, Gallagher MW, Rodriguez P, Schnurr PP, Keane TM. Psychometric properties of the PTSD checklist for diagnostic and statistical manual of mental disorders–fifth edition (PCL-5) in veterans. Psychol Assess. 2016;28(11):1379. 10.1037/pas0000254 26653052

[27] Deane FP, Leathern J, Spicer J, Deane FP, Leathern J, Spicer J (1992). Clinical norms, reliability and validity for the Hopkins Symptom Checklist-21. Aust J Psychol.

[28] Cohen S, Kamarck T, Mermelstein R. A global measure of perceived stress. J Health Soc Behav. 1983;1:385–96. 6668417

[29] Hawthorne G, Herrman H, Murphy B (2006). Interpreting the WHOQOL-BREF: preliminary population norms and effect sizes. Soc Indic Res.

[30] Bowie CR, Harvey PD (2006). Administration and interpretation of the Trail Making Test. Nat Protoc.

[31] Woods SP, Delis DC, Scott JC, Kramer JH, Holdnack JA. The California Verbal Learning Test–second edition: Test-retest reliability, practice effects, and reliable change indices for the standard and alternate forms. Arch Clin Neuropsychol. 2006;21(5):413–20. 10.1016/j.acn.2006.06.002 16843636

[32] Hartman DE (2009). Test Review Wechsler Adult Intelligence Scale IV ( WAIS IV ): return of the gold standard.

[33] Pitman RK, Scott P, Forgue DF, De Jong JB, Claiborn JM (1987). Psychophysiologic assessment of posttraumatic stress disorder imagery in Vietnam combat veterans. Arch Gen Psychiatry.

[34] de Geus EJC, Willemsen GHM, Klaver CHAM, van Doornen LJP (1995). Ambulatory measurement of respiratory sinus arrhythmia and respiration rate. Biol Psychol.

[35] Giuliano RJ, Karns CM, Bell TA, Skowron EA, Neville HJ, Pakulak E (2018). Parasympathetic and sympathetic activity are associated with individual differences in neural indices of selective attention in adults. Psychophysiology..

[36] De Geus EJ, Van Doornen LJ. Ambulatory assessment of parasympathetic/sympathetic balance by impedance cardiography. Ambulatory assessment: Computer-assisted psychological and psychophysiological methods in monitoring and field studies. 1996:141–63.

[37] Kosák M, Hána V, Hill M, Simunkova K, Lacinová Z, Krsek M, Marek J. Serum cortisol seems to be a more appropriate marker for adrenocortical reserve evaluation in ACTH test in comparison to salivary cortisol. Physiol Res. 2014;63(2):229. 10.33549/physiolres.932611 24397810

[38] Behnsen P, Buil M, Koot S, Huizink A, van Lier P (2018). Classroom social experiences in early elementary school relate to diurnal cortisol levels. Psychoneuroendocrinology.

[39] Sztajzel J (2004). Heart rate variability: a noninvasive electrocardiographic method to measure the autonomic nervous system. Swiss Med Wkly.

[40] Kim GM, Woo JM (2011). Determinants for heart rate variability in a normal Korean population. J Korean Med Sci.

[41] Glasofer DR, Brown AJ, Riegel M, Wade T (2015). Structured Clinical Interview for DSM-IV (SCID). Encycl Feed Eat Disord.

[42] Arjadi R, Nauta MH, Scholte WF, Hollon SD, Chowdhary N, Suryani AO (2016). Guided Act and Feel Indonesia (GAF-ID) - Internet-based behavioral activation intervention for depression in Indonesia: study protocol for a randomized controlled trial. Trials.

[43] Blevins CA, Weathers FW, Davis MT, Witte TK, Domino JL. The posttraumatic stress disorder checklist for DSM‐5 (PCL‐5): Development and initial psychometric evaluation. J Trauma Stress. 2015;28(6):489–98. 10.1002/jts.22059 26606250

[44] Asti G. De Psychometrische Eigenschappen van de Indonesische Versie van de PTSD Checklist for DSM-5 ( PCL-5-Ind ) in Javaanse Populatie. Unpubl. master’s thesis: Radboud Univ. Nijmegen: Radboud Universiteit Nijmegen; 2015.

[45] Gray MJ, Litz J, Hsu JL, Lombardo T (2004). Psychometric Properties of the Live Eent Checklist. Assessment.

[46] Turnip SS, Hauff E (2007). Household roles, poverty and psychological distress in internally displaced persons affected by violent conflicts in Indonesia. Soc Psychiatry Psychiatr Epidemiol.

[47] Larson-stoa D, Jacobs GA, Jonathan A (2015). Effect of counseling by paraprofessionals on depression , anxiety , somatization , and functioning in Indonesian torture survivors. Torture..

[48] Smith KJ, Emerson DJ (2014). An assessment of the psychometric properties of the perceived stress Scale-10 (PSS10) with a U.S. public accounting sample. Adv Account.

[49] Elsanti D, Sumarmi N. The effect of stress and social support among postpartum depression women in Indonesia. GSTF J Nursing Health Care (JNHC). 2016;3(2).

[50] Purba FD, Hunfeld JA, Iskandarsyah A, Fitriana TS, Sadarjoen SS, Passchier J, Busschbach JJ. Quality of life of the Indonesian general population: Testretest reliability and population norms of the EQ-5D-5L and WHOQOL-BREF. PLoS One. 2018;13(5):e0197098. 10.1371/journal.pone.0197098 PMC5947896 29750806

[51] Salim OC, Sudharma NI, Kusumaratna RK, Hidayat A (2007). Validity and reliability of World Health Organization Quality of Life-BREF to assess the quality of life in the elderly. Universa Med.

[52] Webber TA, Soble JR (2018). Utility of various WAIS-IV Digit Span indices for identifying noncredible performance validity among cognitively impaired and unimpaired examinees. Clin Neuropsychol.

[53] Suwartono C, Halim MS, Hidajat LL, Hendriks MPH, Kessels RPC (2014). Development and Reliability of the Indonesian Wechsler Adult Intelligence Scale — Fourth Edition (WAIS-IV). Psychology..

[54] Suwartono C, Hidajat LL, Halim MS, Hendriks MPH, Kessels RPC (2016). External Validity of the Indonesian Wechsler Adult Intelligence Scale – Fourth Edition (WAIS-IV-ID). Anima Indones Psychol J.

[55] Egeland J (2015). Measuring working memory with digit span and the letter-number sequencing subtests from the WAIS-IV: too low manipulation load and risk for underestimating modality effects. Appl Neuropsychol.

[56] Delis DC, Freeland J, Kramer JH, Kaplan E, Brandt J, Crosson B (1988). Integrating clinical assessment with cognitive neuroscience: construct validation of the California Verbal Learning Test. J Consult Clin Psychol.

[57] Elwood RW. The California Verbal Learning Test: psychometric characteristics and clinical application. Neuropsychol Rev. 1995;5(3):173–201. 10.1007/BF02214761 8653108

[58] Estiasari R, Fajrina Y, Lastri DN, Melani S, Maharani K, Imran D, Pangeran D, Sitorus F. Validity and reliability of brief international cognitive assessment for multiple sclerosis (BICAMS) in Indonesia and the correlation with quality of life. Neurol Res Int. 2019;2019. 10.1155/2019/4290352 PMC6556319 31263596

[59] Corrigan JD, Hinkeldey NS (1987). Relationships between parts A and B of the trail making test. J Clin Psychol.

[60] Smith SR, Servesco AM, Edwards JW, Rahban R, Barazani S, Nowinski LA (2008). Exploring the validity of the comprehensive trail making test. Clin Neuropsychol.

[61] Wang RY, Zhou JH, Huang YC, Yang YR (2018). Reliability of the Chinese version of the Trail Making Test and Stroop Color and Word Test among older adults. Int J Gerontol.

[62] Shapiro F, Maxfield L. Eye movement desensitization and reprocessing (EMDR): Information processing in the treatment of trauma. J Clin Psychol. 2002;58(8):933–46. 10.1002/jclp.10068 12115716

[63] Colvonen PJ, Glassman LH, Crocker LD, Buttner MM, Orff H, Schiehser DM (2017). Neuroscience and biobehavioral reviews pretreatment biomarkers predicting PTSD psychotherapy outcomes: a systematic review. Neurosci Biobehav Rev.

[64] Coubard OA (2016). An integrative model for the neural mechanism of eye movement desensitization and reprocessing (EMDR). Front Behav Neurosci.

[65] Muller KE, Edwards LJ, Simpson SL, Taylor DJ. Statistical tests with accurate size and power for balanced linear mixed models 2007;3639–3660. 10.1002/sim.2827 17394132

[66] Farina B, Imperatori C, Quintiliani MI, Castelli Gattinara P, Onofri A, Lepore M (2015). Neurophysiological correlates of eye movement desensitization and reprocessing sessions: preliminary evidence for traumatic memories integration. Clin Physiol Funct Imaging.

