# Supplementary material for: Eye Movement Desensitization (EMD) to reduce posttraumatic stress disorder-related stress reactivity in Indonesia PTSD patients: a study protocol for a randomized controlled trial
Source: Trials. 2021 Mar 4;22:181. doi: 10.1186/s13063-021-05100-3 (PMC7931595; doi:10.1186/s13063-021-05100-3)
Supplement: Supplementary file 2 — Additional file 2. [file 13063_2021_5100_MOESM2_ESM.docx]

**APPROVAL-1 STATEMENT (PSP)**

**FOR PARTICIPATION IN RESEARCH SELECTION**

**(INFORMED CONSENT)**

I have obtained an explanation, fully realized, understood, and understood the purpose of screening (selection) of research, and have been given the opportunity to ask questions and have been answered satisfactorily, I can also at any time resign from participation, so I agree / disagree * ) participated in the selection of this study, entitled:

**Eye Movement Desensitization (EMD) to reduce posttraumatic stress disorder-related stress reactivity; a study protocol for a randomized controlled trial**

I voluntarily chose to participate in this research selection without any pressure / coercion.

I agree:

Following the selection as a research participant to ensure the eligibility of post-traumatic stress disorder.

|  | **Day/Date** | **Signiture** |
| --- | --- | --- |
| Name of participant :  Age :  Address : |  |  |
| Researcher : Eka Susanty.,M.Si,M.Psi |  |  |
| Tester : |  |  |

*)cross the unnecessary ones

**RESEARCH INFORMATION**

**TITLE:**

Eye Movement Desensitization (EMD) to reduce posttraumatic stress disorder-related stress reactivity; a study protocol for a randomized controlled trial

**Purpose:**

1. To test whether EMD treatment is more effective than similar treatments but without the component of eye movement (retrieval only) in reducing stress reactivity in patients.

2. To test whether the results of EMD treatments give more positive changes in stress reactions and neuroendocrine activity associated with a decrease in PTSD symptoms compared to similar treatments without the component of eye movement (retrieval only).

**Why You Are Chosen**:

You are chosen to be included in this study because you are in accordance with the criteria that meet the criteria of post-traumatic stress symptoms and are at least 18 years old.

**Procedure:**

If you are willing, the researcher will carry out several stages and actions.

First, the stage of measuring psychological and physiological conditions before EMD treatment is given. At this stage, you will be given a number of questionnaires to find out more in detail about the trauma symptoms that you have experienced. Then the physiological measurements of the heart rate as well as the physiological response to the frequency of the functions of the autonomic nervous system. Physiological measurements are carried out through the skin surface using an ecocardiograph (ECG) device, where seven electrodes will be attached to the surface of the skin at a predetermined point. You will not feel the slightest pain, just a cold feeling because before the skin will be cleaned with alcohol so that the surface of the skin is not obstructed by dirt attached to the surface of the skin. Attaching the electrodes is completely painless and harmless. If you feel uncomfortable removing clothes, the researcher assistant provides a special space for the process of attaching the electrodes and can then re-use the upper garment (T-shirt) during the inspection process.

After the electrodes are installed, you will be asked to listen to the script record related to your traumatic event experienced. When you shows a reaction of anxiety or discomfort the psychotherapist will immediately take over the condition so that it is returned again as before. You will be given emotional relaxation/stabilization to provide emotional stability/calmness. Then a counseling session is conducted to follow up the relaxation/stabilization techniques that have been carried out. After recording the ECG, you will also be informed about the collection of saliva four times which can be done at home. Saliva samples were taken as much as 5 ml using a special pipette and saliva was taken 4 times, when you wake up, half an hour later, noon at 12 and at 4 pm. This initial measurement process takes about 90 minutes.

Next to the intervention stage, you get therapy which will be given directly by a psychotherapist. The process of therapy will last for a maximum of 60 minutes. Each participant will receive a minimum of 4 sessions and a maximum of 6 sessions therapy, the time span between sessions is about 1 week. After the last session, the 4th session or the maximum of the 6th session, the psychological measurements will be taken again using questionnaires, physiological measurements of heartbeats and cortisol sampling, to see the therapeutic effect.

In the final stage, measurements will be taken again 1 month and 3 months after treatment to evaluate the long-term effects of the treatments given.

**Risks and Discomforts**:

In this study, you will feel discomfort or anxiety from the hearing stimulation of the traumatic script. To minimize this, psychotherapists will help in overcoming condition to be as usual. In this study, two types of treatments are carried out namely the EMD treatment that would be given to the experimental group and similar treatments but there are components (Eye Movement) that are removed from the EMD (exposure only) that would be given to the control group. Which group will be given to you, adjusted to the results of randomization (randomization) that has been done.

**Benefits**:

The advantage that can be obtained from this research is the post-traumatic stress symptoms will be reduced or lighter. Services and the role of you are important in this study. This discovery will also help to better understand the mechanism of posttraumatic stress disorder or PTSD (which is more deeply known through the physiological aspects of measurement, namely using echocardiograph)

**Alternative procedure**:

There is no alternative procedure

**Confidentiality Data**:

As long as your participation in this research, any information and data from this research will be treated secrets for others. Your personal data can only be accessed by researchers that involved in this study. Only researchers and official assistants involved in research know the password to enter the data system. The research team will process the data through supervision of the principal investigator.

**Estimated Number of Subjects To be Included**:

94 participants, participants age is at least 18 years

**Volunteerism**:

Your participation in this study voluntary responsibilities until the completion of this study.

**Subject Can Be Excluded / Resigned From Research**:

You freely reject to participate in this study. If you have decided to participate, you can also resign without worrying about anything that is detrimental to you. If you did not follow and fulfill the procedures that are given by the researcher, your participation in this study will end.

**Possible Side Effects**:

In this study, it is very small side effect. If side effects occur and there must be treated, then you will guarantee to free for all costs of psychological care and treatment.

**Complications and Compensation:**

All laboratory examination costs associated with this research will be borne by the researcher. If there are complications or complications related to this study or due to stimulation and psychotherapy, you will be given help with standard agreed procedures and the costs will be borne by this study.

**Contact Person** :

Eka Susanty, Ujung Berung Indah Complex Block 15-11, Bandung, 40611. Tel 022-7804268. HP +6281296164939 or WA +31682311243. E-Mail: [eka.susanty@gmail.com](mailto:eka.susanty@gmail.com)

**APPROVAL-2 STATEMENT (PSP)**

**FOR PARTICIPATION IN RESEARCH TRIAL**

**(INFORMED CONSENT)**

I have read or accepted an explanation, fully understood, and understood the purpose, benefits, and risks that may arise in the research, and have asked for opportunities to ask questions and have answered satisfactorily, as well as the times when I can withdraw from participation, then I (agree / disagree *) participate in this research, titled:

**Eye Movement Desensitization (EMD) to reduce posttraumatic stress disorder-related stress reactivity; a study protocol for a randomized controlled trial**

I voluntarily chose to participate in this research without the pressure /coercion to try. I will be given this permission and the approval sheet that I have signed for my documentation.

I agree:

I am willing to undergo an ECG (echocardiograph) test to measure physiological response related to traumatic event that I have experienced. I am willing to take my saliva sample for a cortisol examination related to my stress condition. I am also ready to be given therapy to reduce post-traumatic stress symptoms (Yes / No *)

|  | **Day/Date** | **Signiture** |
| --- | --- | --- |
| Name of participant :  Age :  Address : |  |  |
| Researcher : Eka Susanty.,M.Si,M.Psi |  |  |
| Tester : |  |  |

*) cross the unnecessary ones
