# Supplementary material for: Eye Movement Desensitization (EMD) to reduce posttraumatic stress disorder-related stress reactivity in Indonesia PTSD patients: a study protocol for a randomized controlled trial
Source: Trials. 2021 Mar 4;22:181. doi: 10.1186/s13063-021-05100-3 (PMC7931595; doi:10.1186/s13063-021-05100-3)
Supplement: Supplementary file 3 — Additional file 3. [file 13063_2021_5100_MOESM3_ESM.doc]

Spirit list 11 C

Protocol : Saliva Samples Collection

Executor: research assistant

Material:

- Saliva collection instruction sheet

- 4 saliva sample tubes

- cooler box

Instructions :

*This instruction sheet is guidance for taking saliva that you are going to do at home. Please take it home to be a guide in taking saliva. Saliva sample collection is carried out four times, ie when you wake up (6 am), the next half hour, 12 noon and 8 pm. I will briefly explain how to do saliva sampling.*

The examiner explains verbally to participants how to collect saliva. After participants understand about instruction, participants are allowed to go home. The instruction sheet is brought home by the participant.

*Is anything unclear? I need to remember that after collecting saliva, the sample tube must be stored in the refrigerator (freezer) at a temperature of around -20. We will take the saliva sample to your house for analysis in the laboratory. Therefore, our permission will contact you tomorrow to take a saliva sample.*
