# Supplementary material for: Eye Movement Desensitization (EMD) to reduce posttraumatic stress disorder-related stress reactivity in Indonesia PTSD patients: a study protocol for a randomized controlled trial
Source: Trials. 2021 Mar 4;22:181. doi: 10.1186/s13063-021-05100-3 (PMC7931595; doi:10.1186/s13063-021-05100-3)
Supplement: Supplementary file 4 — Additional file 4. [file 13063_2021_5100_MOESM4_ESM.doc]

Participant ID :

Session/Date :

Terapist ID :

| **Tahapan** | **Observation /Important note** |
| --- | --- |
| *Life history (sesi -1) /Reevaluation* | Traumatic experience |
| *Preparation* |  |
| *Assessment* | Target Memory target :  SUD :  VOC :  Body scan : |
| *Desensitization* | Number of cycle EM :  SUD :  Stabilization technique: |
| *Closure* |  |
| *Description of therapy process* |  |
