# Supplementary material for: Eye Movement Desensitization (EMD) to reduce posttraumatic stress disorder-related stress reactivity in Indonesia PTSD patients: a study protocol for a randomized controlled trial
Source: Trials. 2021 Mar 4;22:181. doi: 10.1186/s13063-021-05100-3 (PMC7931595; doi:10.1186/s13063-021-05100-3)
Supplement: Supplementary file 5 — Additional file 5. [file 13063_2021_5100_MOESM5_ESM.doc]

| **NO** | **Step** | **Activities** | **Duration** | **Time** | |
| --- | --- | --- | --- | --- | --- |
|  |  |  |  | **Start** | **Stop** |
| 1 | Baseline | Relax | 5’ | 🞏 ........... |  |
|  |  |  |  |  | 🞏 ........... |
| 2 | Neutral Script | Listening neutral script | 3’ | 🞏 ............ |  |
|  |  | Remaining time | 2’ |  | 🞏 ............ |
| 3 | Recovery-1 | Relax  Reading magazines | 5’ | 🞏 ............. |  |
|  |  |  |  |  | 🞏 ............ |
| 4 | Traumatic script | Listening traumatic script | 3’ | 🞏 ............. |  |
|  |  | Remaining time | 2’ |  | 🞏 ............. |
| 5 | Recovery-2 | Relax  Reading magazines | 5’ | 🞏 ............. |  |
|  |  |  |  |  | 🞏 ............. |

Observasi

| **No** | **Jam** | **Deskripsi** |
| --- | --- | --- |
|  |  |  |
