# Supplementary material for: Eye Movement Desensitization (EMD) to reduce posttraumatic stress disorder-related stress reactivity in Indonesia PTSD patients: a study protocol for a randomized controlled trial
Source: Trials. 2021 Mar 4;22:181. doi: 10.1186/s13063-021-05100-3 (PMC7931595; doi:10.1186/s13063-021-05100-3)
Supplement: Supplementary file 6 — Additional file 6. [file 13063_2021_5100_MOESM6_ESM.doc]

Supervision Guideline :

To do

- 1. Therapists carry out therapy process refers to the therapeutic procedures and protocols that have been established by researchers
  2. Provide therapy process for 4 to 6 sessions on the agreed schedule according to the procedure and report the results to researchers
  3. Reporting events that have the potential to interfere with the success of the therapeutic process to researchers (harms or adverse events)
  4. Therapist could be discontinue intervention if the process will be worsening participant condition.
  5. Ensure the security and confidentiality of respondent information in the implementation of the therapy process.
  6. Complete all therapy sessions for each participant being treated

Do not to do (prohibited)

a. Stop as a therapist to provide intervention unilaterally without coordinating with researchers

b. Carry out the therapy process outside the procedures and protocols that have been given

c. Making agreements with participants outside the therapy session

d. Inform and disseminate the confidentiality of personal data of participants outside the research interests
