# Supplementary material for: Eye Movement Desensitization (EMD) to reduce posttraumatic stress disorder-related stress reactivity in Indonesia PTSD patients: a study protocol for a randomized controlled trial
Source: Trials. 2021 Mar 4;22:181. doi: 10.1186/s13063-021-05100-3 (PMC7931595; doi:10.1186/s13063-021-05100-3)
Supplement: Supplementary file 7 — Additional file 7. [file 13063_2021_5100_MOESM7_ESM.doc]

| **Data category** | **Information** |
| --- | --- |
| Primary registry and trial identifying number | ISRCTN : 55239132 |
| Date of registration in primary registry | 19 December 2017 |
| Secondary identifying numbers | NA |
| Source(s) of monetary or material support | Indonesian Endowment Fund for Education (LPDP) Ministry of Research Technology and Higher Education cooperate with Ministry of Finance, Republic of Indonesia through Beasiswa Unggulan Dosen Indonesia (BUDI LN) |
| Primary sponsor | Indonesian Endowment Fund for Education (LPDP) Ministry of Research Technology and Higher Education cooperate with Ministry of Finance, Republic of Indonesia through Beasiswa Unggulan Dosen Indonesia (BUDI LN) |
| Secondary sponsor(s) | NA |
| Contact for public queries | Eka Susanty, [e.s.susanty@vu.nl](mailto:e.s.susanty@vu.nl) |
| Contact for scientific queries | Eka Susanty, [e.s.susanty@vu.nl](mailto:e.s.susanty@vu.nl), Department of Clinical, Neuro and Developmental Psychology, Faculty of Behavioral and Movement Science, Vrije Universiteit Amsterdam, Van der Boechorststraat 7, 1081 BT Amsterdam, The Netherlands |
| Public title | Eye Movement Desensitization (EMD) to reduce posttraumatic stress disorder-related stress reactivity in Indonesia PTSD patients |
| Scientific title | Eye Movement Desensitization (EMD) to reduce posttraumatic stress disorder-related stress reactivity in Indonesia PTSD patients; a study protocol for a randomized controlled trial |
| Countries of recruitment | Indonesia |
| Health condition(s) or problem(s) studied | Posttraumatic Stress Disorder |
| Intervention(s) | Eye Movement Desensitization (EMD) and  Eye Movement Desensitization (EMD) without eye movements |
| Key inclusion and exclusion criteria | Inclusion criteria: diagnosis of PTSD, and adults patient (≥ 18).  Exclusion criteria: current or previous psychotic disorder, current substance use disorder, acute suicidality, and current organic disorder i.e.: epileptic, brain damage |
| Study type | Interventional Allocation: randomized Intervention model: parallel assignment Masking: single blind (assessor) Primary purpose: to test whether particularly Eye Movement Desensitization (EMD), the first part of EMDR treatment, is more effective in reducing stress reactivity in PTSD patients, as compared to a retrieval-only control condition |
| Date of first enrolment | 21 April 2019 |
| Target sample size | 92 |
| Recruitment status | Recruiting |
| Primary outcome(s) | Heart Rate Variability (HRV) |
| Key secondary outcomes | PTSD symptom, Depression and Anxiety symptom, Neurocognitive functioning (memory, attention, executive function, and quality of life). |
