# Supplementary material for: Eye Movement Desensitization (EMD) to reduce posttraumatic stress disorder-related stress reactivity in Indonesia PTSD patients: a study protocol for a randomized controlled trial
Source: Trials. 2021 Mar 4;22:181. doi: 10.1186/s13063-021-05100-3 (PMC7931595; doi:10.1186/s13063-021-05100-3)
Supplement: Supplementary file 8 — Additional file 8. [file 13063_2021_5100_MOESM8_ESM.docx]

**APPROVAL-1 STATEMENT (PSP)**

**FOR PARTICIPATION IN RESEARCH SELECTION**

**(INFORMED CONSENT)**

I have obtained an explanation, fully realized, understood, and understood the purpose of screening (selection) of research, and have been given the opportunity to ask questions and have been answered satisfactorily, I can also at any time resign from participation, so I agree / disagree * ) participated in the selection of this study, entitled:

**Eye Movement Desensitization (EMD) to reduce posttraumatic stress disorder-related stress reactivity; a study protocol for a randomized controlled trial**

I voluntarily chose to participate in this research selection without any pressure / coercion.

I agree:

Following the selection as a research participant to ensure the eligibility of post-traumatic stress disorder.

|  | **Day/Date** | **Signiture** |
| --- | --- | --- |
| Name of participant :  Age :  Address : |  |  |
| Researcher : Eka Susanty.,M.Si,M.Psi |  |  |
| Tester : |  |  |

*)cross the unnecessary ones

**APPROVAL-2 STATEMENT (PSP)**

**FOR PARTICIPATION IN RESEARCH TRIAL**

**(INFORMED CONSENT)**

I have read or accepted an explanation, fully understood, and understood the purpose, benefits, and risks that may arise in the research, and have asked for opportunities to ask questions and have answered satisfactorily, as well as the times when I can withdraw from participation, then I (agree / disagree *) participate in this research, titled:

**Eye Movement Desensitization (EMD) to reduce posttraumatic stress disorder-related stress reactivity; a study protocol for a randomized controlled trial**

I voluntarily chose to participate in this research without the pressure /coercion to try. I will be given this permission and the approval sheet that I have signed for my documentation.

I agree:

I am willing to undergo an ECG (echocardiograph) test to measure physiological response related to traumatic event that I have experienced. I am willing to take my saliva sample for a cortisol examination related to my stress condition. I am also ready to be given therapy to reduce post-traumatic stress symptoms (Yes / No *)

|  | **Day/Date** | **Signiture** |
| --- | --- | --- |
| Name of participant :  Age :  Address : |  |  |
| Researcher : Eka Susanty.,M.Si,M.Psi |  |  |
| Tester : |  |  |

*) cross the unnecessary ones
